# 7 topics that business ecosystems navigate: Assessment of scientific activity and future research agenda

**DOI:** 10.1016/j.heliyon.2023.e16667

**Published:** 2023-05-25

**Authors:** Lorena C. Espina-Romero, Jesús M. Guerrero-Alcedo, Carlos Ossio

**Affiliations:** a Escuela de Postgrado, Universidad San Ignacio de Loyola, 15024 Lima, Peru; b Carrera de Psicología, Universidad Científica del Sur, Lima, Peru

**Keywords:** Enterprise architectures, Identity, Electromobility, Blockchain, Dynamic capabilities

## Abstract

Business Ecosystems are made up of a group of companies that cooperate with each other to innovate in a product. This research had the purpose of assessing the scientific activity and thus be able to extract the topics explored by the authors involved, geographical gaps of scientific production and research topics for future studies. In the methodological design, the statistical software VOSviewer and RStudio were used, with which the documentation obtained from Scopus was analyzed and reflected in tables and figures. The results yielded: a) the 7 topics most worked on by researchers on the variable in question, b) a notable geographical scientific gap in Africa, and c) 5 topics of research that can be explored for future scientific papers. In short, this research was performed with high citation documents, therefore, authors are recommended to carry out research in collaboration with authors located in the geographical scientific gap or vice versa but based on the 5 topics of future research, in the niche topics and a declining topic.

## 1 Introduction

Business ecosystems are the set of organizations made up of government agencies, customers, suppliers, competitors, distributors, among others, which manage to cooperate at the time of a delivery of product and/or service ordered by resorting to competition. Organizations achieve innovation through collaborations with adjunct organizations; this is how companies move beyond their traditional habitat forming organizations with different interests where they interact thanks to the Internet, creating opportunities for innovation and new challenges for well-established companies [1]. Innovative business ecosystems or also the so-called disruptive business models, are specific environments where a set of elements converge such as the availability of talented professionals, generation of knowledge, stakeholders, proximity to suppliers, among others [2].

Within a business ecosystem there are well-established companies where talented executives are trained, who are the ones who make the decisions when mobilizing markets [3]. Currently, digital transformation encompasses the entire business ecosystem, which is necessary for the prosperity of any business. Digital media, technology, and ways of doing business play an key role during the development of companies, and the advent of the Internet of Things that helps in the efficiency of all processes, with the collection of information quickly facilitating decision-making for any situation [4].

There are many topics that business ecosystems navigate [5], but to keep up to date with the scientific activity related to this variable and its impact on research and society, it is inevitable to ask, “what are the associated topics that business ecosystems navigate?” The answer will be obtained through a bibliometric and bibliographic analysis to interpret the data generated in the topic, summary of main data, research topics worked by researchers, scientific production by country, geographical scientific gap and, finally, an agenda for future research.

The general purpose of this research is to assess the scientific activity linked to business ecosystems during the period 2017–2021, and thus be able to extract the topics explored by the researchers involved, geographical scientific gaps and research topics for future studies; all under two criteria, bibliometric and bibliographic.

## 2 Methodology

This research takes into consideration the application of a bibliometric review, and the steps formulated by Ivan Zupic and Tomaz Cater have been maintained: the identification of the research design, the collection, the data analysis, visualization, and interpretation [6]. Detailed information was obtained from the Scopus database through the search string: TITLE (“BUSINESS ECOSYSTEM” OR “BUSINESS ECOSYSTEMS”) AND (LIMITED TO (PUBYEAR, 2021) OR LIMITED TO (PUBYEAR, 2020) OR LIMITED TO (PUBYEAR, 2019) OR LIMITED TO (PUBYEAR, 2018) OR LIMIT A (PUBYEAR, 2017)). Then, a quantitative assessment was applied to the data obtained, within a bibliometric approach on the variable business ecosystems, as well as a qualitative assessment of the documents of the authors selected for this research but using at the same time a bibliographic approach to obtain their perceptions regarding the subject under study.

Based on the steps proposed by Ivan Zupic and Tomaz Cater, it was decided to divide the methodology applied in this bibliometric review into 3 phases. Starting with Phase 1, where data was collected through the Scopus platform introducing the variable under study and resulting in 234 documents published during the period 2017–2021; it should be noted that there was no limitation by type of document selected by the authors at the time of publication, no country was left out and all the subject areas involved with the variable in question were accepted.

In Phase 2 the analysis materials were organized, the data from the files in RIS and BibTex format were loaded to the VOSviewer software version 1.6.18 and RStudio in its version 4.1.1, which generated figures and tables because of the evaluation of the data obtained from Scopus. These figures and tables were interpreted and then analyzed a) the summary of the main data, b) the topics most explored by the researchers with the variable under study, c) scientific production by country together with the geographical scientific gap and d) an agenda for future research was deduced. Finally, in Phase 3, the drafting of the conclusions and the elaboration of the final document were carried out to comply with the general objective of this research.

### 2.1 Methodological design

Table 1 describes and classifies the three phases that make up the methodological design applied in this research.

**Table 1:** Methodological design.

	Phases	Description	Classification
PHASE 1	DATA COMPILATION	234 documents were profiled that make up the data obtained using the search tool of the Scopus platform.	Indexed publications with the variable Business ecosystems. Documents published during the period 2017–2021. All types of publication are recorded, no country is excluded. Without distinction in the subject areas.
PHASE 2	CONSTRUCTION OF ANALYSIS MATERIAL	The documentation outlined in Phase 1 was organized either by means of tables and figures supported by the information collected from Scopus with the help of the VOSviewer version 1.6.17 and RStudio 4.1.1 software.	Summary of main data. Research topics worked on by researchers. Scientific production by country. Geographical scientific gap. Agenda for future research.
PHASE 3	DRAFTING OF CONCLUSIONS, RECOMMENDATIONS AND THE OUTCOME DOCUMENT	Once the analysis carried out in Phase 2 was completed, the conclusions and development of the final document were drafted.	

## 3 Results and discussions

### 3.1 Summary of the main information

Table 2 shows the main data information collected in the Scopus database, starting with the total of 234 documents related to business ecosystems during the period 2017–2021. Each document received 5.96 citations on average. It also shows 533 unique authors who obtained 686 appearances. There are 24 documents by a single author. An average of 2.43 authors collaborated in the realization of each document. The average number of documents per author is 0.44, as well as the average number of authors per document is shown at 2.28, while the average of co-authors per document yielded 2.93. All this information and especially the average citations per document (5.96), allows us to deduce that the manuscripts that have been obtained on the Business Ecosystems are of very good quality and high impact, which will help to meet the general objective set out in this research.

**Table 2:** Summary of main information.

Description	Results
MAIN INFORMATION ABOUT DATA	
Timespan	2017–2021
Sources (Journals, Books, etc.)	159
Documents	234
Average years from publication	2.83
Average citations per documents	5.957
Average citations per year per doc	1.343
References	10333
DOCUMENT TYPES	
article	118
book	2
book chapter	12
conference paper	94
editorial	1
note	1
review	6
DOCUMENT CONTENTS	
Keywords Plus (ID)	989
Author's Keywords (DE)	645
AUTHORS	
Authors	533
Author Appearances	686
Authors of single-authored documents	22
Authors of multi-authored documents	511
AUTHORS COLLABORATION	
Single-authored documents	24
Documents per Author	0.439
Authors per Document	2.28
Co-Authors per Documents	2.93
Collaboration Index	2.43

### 3.2 Research topics with the variable business ecosystems

**Fig. 1:**
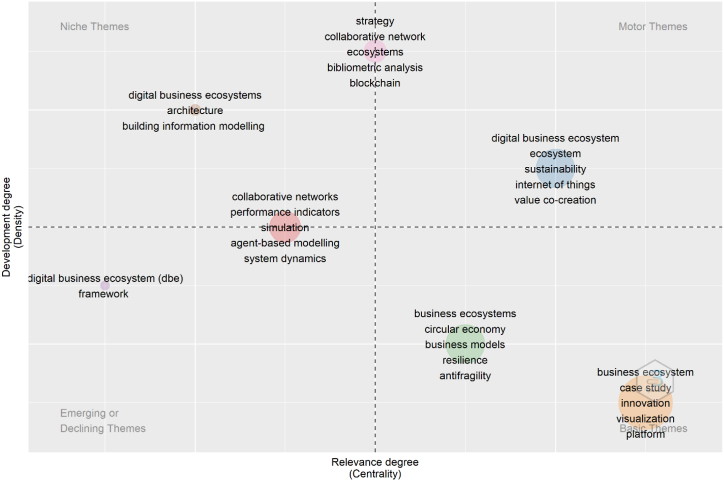
Thematic map of research flows.

The information obtained from the Scopus database, particularly the author keywords (645), was uploaded to the RStudio software using the Thematic Map tool to organize and represent the selected terms and thus be able to form opinions and ideas on the topics of study. Fig. 1 shows the Thematic Map generated by the RStudio software, which shows 7 clusters distributed in the 4 quadrants of the figure. After the bibliometric analysis, a bibliographic analysis of the content was carried out to complete the quantitative and qualitative process, resulting in 7 research themes where the variable Business Ecosystems navigates. These themes are: 1) business innovation ecosystem, 2) circular economy as a business model, 3) sustainable digital businesses, 4) collaborative networks for performance indicators, 5) collaborative network as a strategy for the Blockchain ecosystem, 6) digital business architecture under construction, and 7) digital business ecosystems and co-petition. Table 3 describes the visual information contained in Fig. 1.

**Table 3:** Description, category, and coverage of research themes.

N. Theme	Theme Description	Theme Category	% Coverage in the 234 documents
1	business innovation ecosystem	Basic Themes	51.44
2	circular economy as a business model	Basic Themes	16.19
3	sustainable digital business	Motor Themes	15.67
4	collaborative networks for performance indicators	Emerging Themes	8.88
5	collaborative network as a strategy for the Blockchain ecosystem	Niche Themes	4.44
6	digital business architecture under construction	Niche Themes	1.83
7	digital business ecosystems and co-petition	Declining Themes	1.57

#### 3.2.1 Theme 1: business innovation ecosystem

According to Table 3, this is considered a basic theme and covers 51.44% of the 234 documents selected for this research. This research stream studies the generation of environments where the perspectives, efforts, and potentialities of a group of organizations come together to collaborate in the transformation of knowledge for the benefit of innovation.

Of this current stands out the work entitled “Maintaining superior performance in enterprise ecosystems: Evidence from application software developers in the iOS and Android smartphone ecosystems” [7], where a theoretical framework is developed to understand how the evolutionary and structural particularities of an ecosystem manage to shape to the same extent that complementary companies sustain their performance to the maximum.

Another document related to this topic is “Adaptation and maintenance of operations in weak institutional environments: an evaluation of the business ecosystem of a Chinese multinational company in Central Africa” [8], where the adaptation and maintenance of operations of multinational companies in environments of countries with precarious, challenging, and weak institutions due to the devastation of conflicts is investigated. Such is the case of a Chinese state-owned company that managed to enter and develop operations in the Democratic Republic of the Congo (DRC) located in Central Africa. The findings demonstrated that the Chinese company remained operational and engaged in collective actions, as well as co-evolving with key stakeholders within its own business ecosystem. Another group of documents related to this topic were made by the authors cited below: [[9], [10], [11], [12], [13], [14], [15]].

#### 3.2.2 Theme 2: circular economy as a business model

According to Table 3, this is also considered a basic theme and covers 16.19% of the 234 documents chosen for this research. The research developed for this topic deals with circular business models, which are focused on the reduction of waste while creating value through the continuous circulation of elements, materials, including waste that is the product of the manufacturing and sale process, as well as the conservation and restitution of exploited resources. To carry out this complete process, certain steps must be followed in all areas that make up the circular business model, and these are: repair, reuse, redistribute, recycle, and regenerate.

Among the research related to this topic, is “Governing a sustainable business ecosystem in Taiwan's circular economy: the history of the spring pool glass” [16], that proposes to uncover the total number of mechanisms that a circular economy company applies to the governance of its business ecosystem; a longitudinal case study is applied in a company where glass is recycled in Taiwan; the findings show that the rescued mechanisms include: use of stakeholder networks for the development of the business ecosystem, long-term capture of value to be able to entering emerging markets, corporate social responsibility and brand image, company capacity, research and development in recycling and adaptation to government policy.

Another document is the article entitled “The mechanisms for members of the business ecosystem to capture part of the value created together of an entrepreneurial ecosystem” [17], that studies the tools by which the partners in a business ecosystem, manage to capture value created from a linear open tape ecosystem (LTO) and how these tools contribute to the sustainability of the business ecosystem; three tools were identified that help business ecosystem partners stay within and favor sustainability and their success. Other manuscripts related to the theme of circular economy are by the following authors: [[18], [19], [20], [21], [22]].

#### 3.2.3 Theme 3: sustainable digital business

According to Table 3, this topic is considered a Motor Theme, i.e., main themes of the research front. The documents linked to this trend represent 15.67% of the 234 manuscripts chosen for this research and deal with how digital business ecosystems assume the identity of being a green ecosystem, therefore prioritizing care of the environment framed in a sustainability model.

A document related to this topic is “Expanding organizational capabilities with open data to support sustainable and dynamic business ecosystems” [23], where the authors propose solving challenges related to open data (OD) by creating an business ecosystem for OD, based on a joint network supporting interaction between knowledge management and OD provisioning; a progressive approach was applied supported by a series of capabilities, recognizing the modeling of OD processing ecosystems, enabling the reciprocity of knowledge on the practice of open data among ecosystem partners and allowing information systems to be configured in the OD process.

Another article focused on this topic is entitled “Building sustainable business ecosystems through customer engagement: a lesson from the south Korean cases” [24], where the consequences of customer intervention during the creation of economic and social values within a business ecosystem are examined; with in-depth interviews with several South Korean companies, the study yielded such results as the accumulation of economic and social values for both society and companies. Other documents where this topic was studied are by the following authors: [[25], [26], [27], [28], [29]].

#### 3.2.4 Theme 4: collaborative networks for performance indicators

According to Table 3, this topic is considered as emerging, i.e., research on this topic arises without being foreseen and represents 8.88% of the 234 manuscripts indexed and selected for this research. This topic deals with how the network or interconnected social networks of a company are analyzed for the benefit of organizational management, and then extract the performance indicators that describe the reality of the organization.

A book chapter on this topic is “Exploration of performance evaluation scenarios in collaborative business ecosystems” [30], where performance and influence mechanism are evaluated to promote sustainable collaborative behaviors within a collaborative business ecosystem and to determine performance; to do this, a simulation model was designed assessing the suggested perspective that was then discussed based on the results obtained.

The following article linked to this topic is “Background to co-development and its effect on innovation performance: a perspective of the business ecosystem” [31]; its objective was to analyze a research prototype with a business ecosystem approach, where the core elements of joint growth were identified as well as how these elements influence innovation performance in the hospitality and information and communication technology (TIC) industries in Taiwan; the results of both industries explain that the joint growth of a company under its own ecosystem has effective results in innovative performance. Other articles that study this topic are by the authors: [[32], [33], [34], [35], [36]].

#### 3.2.5 Theme 5: collaborative network as a strategy for the blockchain ecosystem

In Tables 3 and it is considered one of the niche themes and is part of 4.44% of the 234 works selected for this research. This low percentage of documents allows us to infer that this topic has not been well used when investigating business ecosystems. This theme deals with how a collaborative network made up of users can track payments, accounts, orders, among others and share an exclusive source of information that allows creating efficiency and trust in Blockchain ecosystems.

One of the most cited articles is “Improving trust in business ecosystems with Blockchain technology” [37], where the authors describe a Blockchain architecture design process that manages to improve the trust of a given domain, how to avoid traps, lessons learned and best practices; all focused on the benefit of professionals to reduce technological risks and improve trust in Blockchain ecosystems.

Another document related to this topic is “Blockchain in innovation in health care: literature review and case study from a business ecosystem perspective” [38], which aims to explore a paradigm shift and the development of the healthcare ecosystem using Blockchain technology; through a bibliographic analysis with a case study, 4 stages of the life cycle of a business ecosystem made up of birth, expansion, leadership and self-renewal or death were examined, the results showed that the impacts directed at the traditional health industry stand out in the 4 stages favoring a paradigm shift of health in the future. Other research papers linked to this theme were written by the authors: [[39], [40], [41]].

#### 3.2.6 Theme 6: digital business architecture under construction

This is considered as one of the niche themes for research in Table 3 and comprises 1.83% of the 234 documents chosen for this research, which makes us infer how little the construction industry has collaborated to form digital ecosystems to its benefit, while maintaining its analog processes. Therefore, construction remains one of the least digitized industries worldwide, and is due to many peculiarities such as the low technological qualification and the low interest in introducing new working methods in the brief time that a project lasts by suppliers and subcontractors. On the other hand, these companies do not have enough budget for research, development, and innovation.

An indexed manuscript on this subject is the one titled “From the Finnish AEC knowledge ecosystem to the business ecosystem: lessons learned from the national deployment of BIM” [42], where a field study of Finland's AEC architecture, engineering and construction industry resulted in the critical understanding of why nationwide research and development and the adoption of BIM building information modeling technology in Finland have failed to bring the evolution that was expected of its AEC business ecosystem. The findings provide input for BIM researchers and governments to establish new policies on BIM adoption better aligned with systemic evolution related to business practices within the AEC ecosystem.

Another document is the book chapter “The digital business ecosystem as an enabler of eco-innovation in the construction sector” [43], which is aimed at providing information on the development of DBE Digital Business Ecosystems and their role in the transfer of green innovation in the construction industry in Latvia. The results of a survey showed that the concept of DBE is new and supports the combination of 3 networks: a) TIC, b) social networks and c) knowledge networks. In addition to this result, it was shown that the evolution of the DBE in the construction industry is in its initial stage in Latvia, therefore, policy makers must be attentive to the promoters of its further development, as well as with general digital skills, digital infrastructure, education, among others.

#### 3.2.7 Theme 7: digital business ecosystems and coopetition

In Table 3, this is described as a declining theme, i.e., there is an average of 6 documents per year from 2017 to 2020 and only 1 document for the year 2021. It represents 1.57% of the 234 documents allocated for this research. Research on this topic explores digital ecosystems as a system where a group of representatives works collectively under the premise of coopetition, that is, they cooperate under their own and shared rules and infrastructures, in addition to competing independently in the creation and offer of services to their respective clients regardless of their structure and nature.

An investigation with this theme is the work “Innovation process in the business ecosystem: the four practices of cooperation in the media platform” [44], which objective was to develop a group of innovation methods that result from an ecosystem supported by academic research; the over-the-top (OTT) platform is valued under the inspiration of creativity and culture that encompasses a hybrid network of ecosystem partners, all through the use of 4 methods of cooperation. The findings revealed how co-innovative entrepreneurial ecosystems were able to demonstrate coevolution through different directions and structures. Other documents related to this research stream were by the following authors: [[45], [46], [47], [48]].

To summarize this section, these seven topics relate to contemporary business practices and strategies that aim to create more sustainable and socially responsible organizations.

## 4 Scientific production by country and geographical scientific gap

Many countries that have conducted research on business ecosystems seek the path of cooperation, by which research is registered by more than two countries interested in the study variable. Therefore, the total number of manuscripts indicated in the methodological design does not correspond to the total number of documents registered for all the participating countries.

**Fig. 2:**
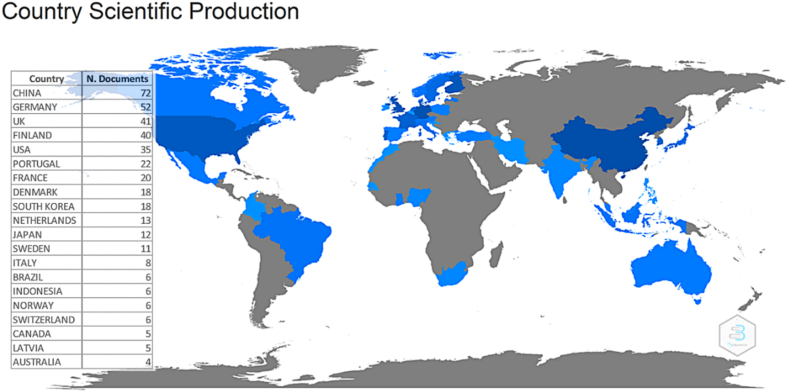
Scientific production by country.

Fig. 2 shows in blue color the countries with document production around Business Ecosystems. China (72) leads the production of documents as they have an adequate business ecosystem due to their attempt to imitate the Silicon Valley model since the 1990s, therefore, they do not depend on external decisions. It is followed in second and third place by Germany (52) and the United Kingdom (41). Finland (40) is fourth, followed by the United States (35) in fifth place. Portugal (22), France (20), Denmark (18), South Korea (18) and the Netherlands (13) are in sixth, seventh, eighth, ninth and tenth place, respectively. The rest of the countries, with fewer documents, can be found in the text box framed Fig. 2.

Fig. 2 also shows in gray color the countries that have not produced research on Entrepreneurial Ecosystems during 2017–2021. Among those are in the American continent countries such as Chile, Argentina, Peru, Ecuador, Paraguay, Uruguay, Bolivia, Venezuela, Guyana, Suriname, Panama, French Guinea, Costa Rica, Nicaragua, Honduras, Guatemala, Belize, Dominican Republic, Haiti, and Cuba. Africa, made up of 54 nations, has the most countries without document production except for South Africa, Nigeria, Ghana, Senegal, and Morocco. From the Middle East, countries without research such as Oman, Yemen, Saudi Arabia, the United Arab Emirates, Jordan, Iraq, and Syria can be seen. From Eurasia Russia and the countries that make up the old Soviet Union are shown. In Asia there are countries such as Pakistan, Nepal, Bangladesh, Bhutan, Burma, Thailand, Laos, Vietnam, Cambodia, Papua New Guinea, and New Zealand. Eastern Europe includes Romania, Bulgaria, Ukraine, Hungary, Czech Republic, Slovakia, Serbia, Poland, Belarus, and Estonia.

It should be noted the considerable number of countries without production of documents on the variable of interest, but the greatest geographical scientific gap is located on the African continent. There are five reasons for the geographical scientific gap on the African continent. First, the lack of investment in research and development by African countries which have limited resources. Second, the lack of international collaboration, i.e., collaboration between scientists from African universities and scientists from other countries is also limited. Third, the digital divide and internet access, which, despite increased connectivity on the continent, there is still a significant digital divide. Fourth, the brain drains, as many highly trained African scientists migrate to other countries in search of better research and job opportunities. Finally, are inadequate government policies to promote scientific research and development on the continent.

## 5 Agenda for future research

**Fig. 3:**
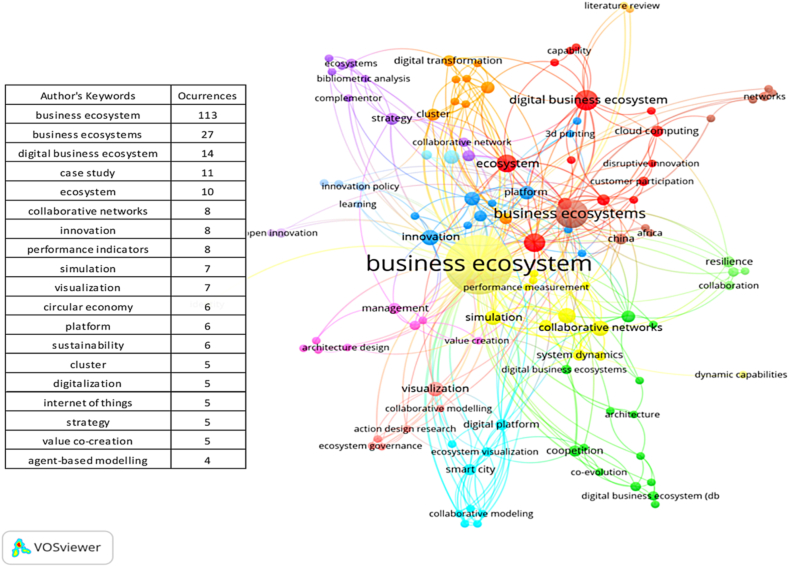
Term map for future research.

**Fig. 4:**
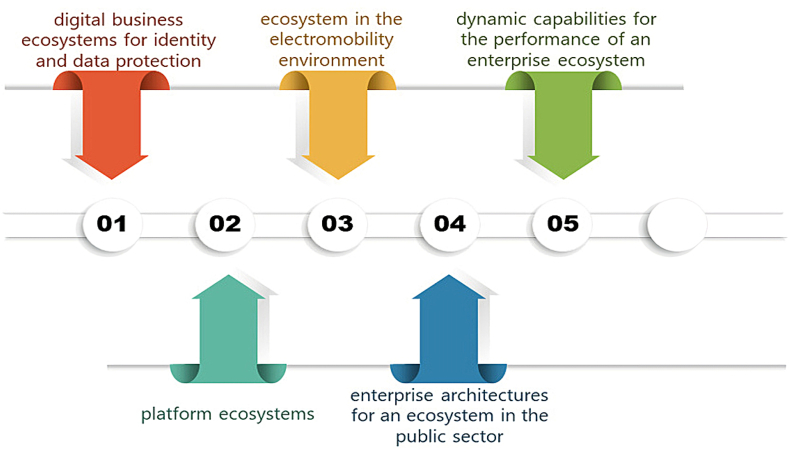
Topics of future research.

Fig. 3 generated by the VOSviewer software, Term Map for future research, allows to deduce topics of research related to the variable under study. After the discussion of the data provided by the Scopus database, including 989 Plus keywords, 645 author keywords and the 234 titles of documents together with their abstracts, 5 topics of exploration were derived that can be subject of future research. Among them: 1) digital business ecosystems for identity and data protection, 2) platform ecosystems, 3) ecosystem in the electromobility environment, 4) enterprise architectures for an ecosystem in the public sector, and 5) dynamic capabilities for the performance of an enterprise ecosystem. These 5 topics are illustrated in Fig. 4.

### 5.1 Digital business ecosystems for identity and data protection

This topic of research addresses access and the security of digital identity, determined by a set of elements in its variety of uses, which calls into question deficiencies during the management of users or external customers who, added to the necessary requirements of data protection and privacy, demand that approaches be investigated to adapt to the new digital era [49].

### 5.2 Platform ecosystems

This topic of research explains how the ecosystems that have evolved the most are those based on digital platforms, because they involve millions of partners and incorporate many digital offers. This type of ecosystem is based on the “data first” strategy to use the knowledge provided by customers for the continuous sale and design of novel offers, thanks to the generation of data. An example of this is Google Home, in which producers, developers and engineers work together to create appliances that link to this platform to be smart and connected [50].

### 5.3 Ecosystem in the environment of electromobility

This topic of research describes what a wide variety of technologies originated by the electromobility ecosystem and aimed at business fleets would mean for many companies the difference between surviving in a competitive market or dying for not adapting to the demanding operation rules [51].

### 5.4 Enterprise architectures for a digital ecosystem in the public sector

This topic of research deals with how the digital technologies of enterprise architectures could allow the creation of an exclusive technological ecosystem, where services can be performed for the public sector, but that must be designed so that it can adapt to the needs of the organization [52].

### 5.5 Dynamic capabilities for the performance of an enterprise ecosystem

This research subject exposes how dynamic capabilities, described as processes, skills, organizational structures, procedures, disciplines, and decision rules, can help to detect, and capture opportunities that emerge in an organization. Therefore, dynamic capabilities allow ecosystems to implement, create and safeguard non-material assets that, in the future, will enable good commercial results [53].

It should be noted that these five topics for future studies are related to technology and its impact on society.

## 6 Conclusions and recommendations

Once the results and discussion have been completed, it should be noted that the 234 documents selected for this research are of quality and high impact as shown by the average number of citations per document (5.96). Fulfilling the first part of the general objective, it was possible to determine 7 research topics linked to the variable under study, where 2 are basic themes, 1 is a motor theme, 2 are niche themes and 1 is a theme in decline. It was also possible to detect a scientific gap from the geographical point of view in terms of the production of studies at a global level, but the most notable is the geographical scientific gap represented by the African continent, thus fulfilling the second part of the general objective. After an analysis of keywords plus, author keywords, titles of documents and their abstracts, 5 topics that can be explored in future research were guaranteed, all linked to the variable business ecosystems, in this way the third part of the general objective is fulfilled. Business ecosystems are a critical topic in business research and are addressed from different approaches. Innovation, circular economy, sustainability, collaborative networks, digital enterprise architecture and coopetition are key topics for the success of these ecosystems.

It is recommended that researchers in general conduct studies on Business Ecosystems linked to identity and data protection, the electromobility environment, digital ecosystems for the public sector and the dynamic capabilities for the proper functioning of these ecosystems. These researchers could carry out these studies with authors from other countries that do not have scientific production in this area, especially with countries on the African continent where the geographical scientific gap is high. Why Africa? This continent has potential thanks to its demographic expansion, technological advances, and an upward demand for quality education; there is an increase in African students enrolled in academic programs abroad. So, it's no wonder that African higher education is in growing demand. Therefore, this demographic push coupled with growing wages presents an option to research in collaboration with this forward-looking generation, willing to invest in education and research and thus improve their standard of living.

This research on business ecosystems has significant implications for how companies interact with their environment and compete in the marketplace. This study highlights that companies that innovate, collaborate, adapt in a timely manner, create a collaborative business culture, and create shared value, perform better within a proactive and competitive business field. This research is updated to January 2022 and the information is limited to a single scientific database called Scopus.

## Author contribution statement

All authors listed have significantly contributed to the development and the writing of this article.

## Data availability statement

Data included in article/supplementary material/referenced in article.

## Additional information

No additional information is available for this paper.

## Declaration of competing interest

The authors declare that they have no known competing financial interests or personal relationships that could have appeared to influence the work reported in this paper.
